# 3-(3-Cyano­phen­yl)-*N*-phenyl­oxirane-2-carboxamide

**DOI:** 10.1107/S1600536810037475

**Published:** 2010-09-25

**Authors:** Tai-Ran Kang

**Affiliations:** a College of Chemistry and Chemical Engineering, China West Normal University, Nanchong 637002, People’s Republic of China

## Abstract

In the title compound, C_16_H_12_N_2_O_2_, both terminal benzene rings are located at the same side of the central epoxide ring, showing a *cis* conformation. The epoxide ring makes dihedral angles of 76.59 (10) and 62.40 (11)° with the phenyl and cyano­phenyl rings, respectively. Inter­molecular N—H⋯O and weak C—H⋯O hydrogen bonding is present in the crystal structure.

## Related literature

For the use of epoxide-containing compounds as building blocks in synthesis, see: Meth-Cohn & Chen (1999 ▶); Porter & Skidmore (2000 ▶); Righi *et al.* (1996 ▶); Thijs *et al.* (1990 ▶). For related structures, see: Chen & Kang (2009*a* ▶,*b* ▶); He (2009 ▶); He *et al.* (2009 ▶).
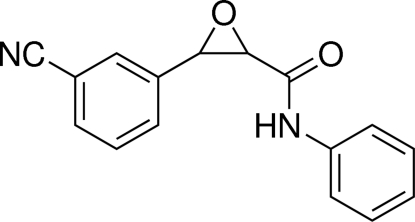


## Experimental

### 

#### Crystal data

C_16_H_12_N_2_O_2_
*M* *_r_* = 264.28
Orthorhombic, 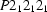

*a* = 5.459 (2) Å
*b* = 11.141 (7) Å
*c* = 21.844 (5) Å
*V* = 1328.6 (10) Å^3^
*Z* = 4
Mo *K*α radiation
μ = 0.09 mm^−1^
*T* = 291 K
0.36 × 0.32 × 0.26 mm

#### Data collection

Oxford Diffraction Gemini S Ultra diffractometer
9766 measured reflections
2078 independent reflections
1378 reflections with *I* > 2σ(*I*)
*R*_int_ = 0.042

#### Refinement

*R*[*F* ^2^ > 2σ(*F* ^2^)] = 0.038
*wR*(*F* ^2^) = 0.072
*S* = 1.00
2078 reflections
181 parameters
1 restraint
H-atom parameters constrained
Δρ_max_ = 0.14 e Å^−3^
Δρ_min_ = −0.18 e Å^−3^

### 

Data collection: *CrysAlis PRO* (Oxford Diffraction, 2009 ▶); cell refinement: *CrysAlis PRO*; data reduction: *CrysAlis PRO*; program(s) used to solve structure: *SHELXS97* (Sheldrick, 2008 ▶); program(s) used to refine structure: *SHELXL97* (Sheldrick, 2008 ▶); molecular graphics: *ORTEP-3* (Farrugia, 1997 ▶); software used to prepare material for publication: *SHELXL97*.

## Supplementary Material

Crystal structure: contains datablocks global, I. DOI: 10.1107/S1600536810037475/xu5025sup1.cif

Structure factors: contains datablocks I. DOI: 10.1107/S1600536810037475/xu5025Isup2.hkl

Additional supplementary materials:  crystallographic information; 3D view; checkCIF report

## Figures and Tables

**Table 1:** Hydrogen-bond geometry (Å, °)

*D*—H⋯*A*	*D*—H	H⋯*A*	*D*⋯*A*	*D*—H⋯*A*
N1—H4⋯O1^i^	0.86	2.46	3.239 (3)	151 (1)
C11—H11⋯O1^i^	0.93	2.56	3.467 (3)	165 (1)
C12—H12⋯O2^ii^	0.93	2.55	3.302 (3)	139 (1)

Symmetry codes: (i) 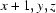; (ii) 
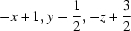
.

**Fig. 1.**
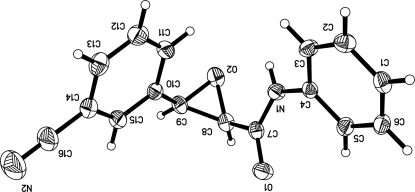
The molecular structure of (I) with 30% probability displacement ellipsoids (arbitrary spheres for H atoms).

## supplementary crystallographic information

### Comment

α, β-epoxides are key intermediates for synthesizing some natural products (Porter & Skidmore, 2000; Righi *et al.*, 1996). Selective ring opening reactions of oxiranes also provide powerful and efficient routes to a variety of useful compounds including 2,3-epoxyketone (Meth-Cohn *et al.*, 1999), aziridinecarboxylate (Thijs *et al.*, 1990). Various effective systems have been developed over the years for the preparation of chiral epoxides. As a part of our interest in the synthsis of epoxides ring systems (Chen & Kang, 2009*a*,*b*; He, 2009, He *et al.* (2009)), we synthesis the title compound by using Darzens reaction. We report herein the crystal structure of the title compound.

The molecular structure of (I) is shown in Fig. 1. Bond lengths and angles in (I) are normal. The cyanophenyl ring and *N*-phenylformamide units adopts a *cis* conformation with respect to the epoxides ring, the dihedral angle between the two phenyl ring is 84.75 (6)°. Epoxide ring makes dihedral angles of 76.59 (10)° and 62.40 (11)° with phenyl rings C1—C6 and C10—C15, respectively. The crystal packing is stabilized by C—H···O and N—H···O hydrogen bonding (Table 1).

### Experimental

2-Chloro-*N*-phenylacetamide (0.17 g, 1.0 mmol) and sodium ethanolate (0.14 g, 2.0 mmol) were dissolved in acetonitrile (2 ml). To the solution was added 3-formylbenzonitrile (0.131 g, 1.0 mmol) at 298 K, the solution was stirred for 60 min and removal of solvent under reduced pressure, the residue was purified through column chromatography on silica gel to give compound (I). Crystals suitable for X-ray analysis were obtained by dissolving the title compound (0.02 g) in ethyl acetate (2 ml) and evaporating the solvent slowly at room temperature for about 1 d.

### Refinement

H atoms were placed in calculated positions, with C—H = 0.93–0.98 Å, and N—H = 0.86 Å, and refined using a riding model with *U*_iso_(H) = 1.2*U*_eq_(C,N). As no significant anomalous scattering, Friedel pairs were merged.

### Crystal data

**Table:**

C_16_H_12_N_2_O_2_	*D*_x_ = 1.321 Mg m^−^^3^
*M**_r_* = 264.28	Mo *K*α radiation, λ = 0.71073 Å
Orthorhombic, *P*2_1_2_1_2_1_	Cell parameters from 3256 reflections
*a* = 5.459 (2) Å	θ = 3.3–29.0°
*b* = 11.141 (7) Å	µ = 0.09 mm^−^^1^
*c* = 21.844 (5) Å	*T* = 291 K
*V* = 1328.6 (10) Å^3^	Block, colorless
*Z* = 4	0.36 × 0.32 × 0.26 mm
*F*(000) = 552	

### Data collection

**Table:**

Oxford Diffraction Gemini S Ultra diffractometer	1378 reflections with *I* > 2σ(*I*)
Radiation source: Enhance (Mo) X-ray Source	*R*_int_ = 0.042
graphite	θ_max_ = 29.2°, θ_min_ = 3.3°
Detector resolution: 15.9149 pixels mm^-1^	*h* = −7→3
ω scans	*k* = −15→15
9766 measured reflections	*l* = −29→27
2078 independent reflections	

### Refinement

**Table:**

Refinement on *F*^2^	Primary atom site location: structure-invariant direct methods
Least-squares matrix: full	Secondary atom site location: difference Fourier map
*R*[*F*^2^ > 2σ(*F*^2^)] = 0.038	Hydrogen site location: inferred from neighbouring sites
*wR*(*F*^2^) = 0.072	H-atom parameters constrained
*S* = 1.00	*w* = 1/[σ^2^(*F*_o_^2^) + (0.0322*P*)^2^] where *P* = (*F*_o_^2^ + 2*F*_c_^2^)/3
2078 reflections	(Δ/σ)_max_ < 0.001
181 parameters	Δρ_max_ = 0.14 e Å^−^^3^
1 restraint	Δρ_min_ = −0.18 e Å^−^^3^

### Special details

**Table:**

Geometry. All e.s.d.'s (except the e.s.d. in the dihedral angle between two l.s. planes) are estimated using the full covariance matrix. The cell e.s.d.'s are taken into account individually in the estimation of e.s.d.'s in distances, angles and torsion angles; correlations between e.s.d.'s in cell parameters are only used when they are defined by crystal symmetry. An approximate (isotropic) treatment of cell e.s.d.'s is used for estimating e.s.d.'s involving l.s. planes.
Refinement. Refinement of *F*^2^ against ALL reflections. The weighted *R*-factor *wR* and goodness of fit *S* are based on *F*^2^, conventional *R*-factors *R* are based on *F*, with *F* set to zero for negative *F*^2^. The threshold expression of *F*^2^ > σ(*F*^2^) is used only for calculating *R*-factors(gt) *etc*. and is not relevant to the choice of reflections for refinement. *R*-factors based on *F*^2^ are statistically about twice as large as those based on *F*, and *R*- factors based on ALL data will be even larger.

### Fractional atomic coordinates and isotropic or equivalent isotropic displacement parameters (Å^2^)

**Table:**

	*x*	*y*	*z*	*U*_iso_*/*U*_eq_	
O2	0.2729 (2)	1.02915 (12)	0.76684 (6)	0.0522 (4)	
O1	−0.2382 (2)	0.87594 (13)	0.69492 (6)	0.0540 (4)	
N1	0.1739 (3)	0.86142 (13)	0.67637 (7)	0.0416 (4)	
H4	0.3135	0.8914	0.6864	0.050*	
C4	0.1740 (3)	0.76926 (15)	0.63124 (8)	0.0350 (4)	
C3	0.3670 (3)	0.68860 (17)	0.63104 (9)	0.0430 (5)	
H3	0.4906	0.6946	0.6602	0.052*	
C6	−0.0019 (4)	0.67021 (17)	0.54450 (8)	0.0453 (5)	
H6	−0.1252	0.6639	0.5153	0.054*	
C10	0.1794 (3)	0.85668 (17)	0.83606 (8)	0.0389 (4)	
C2	0.3746 (4)	0.59914 (18)	0.58733 (9)	0.0481 (5)	
H2	0.5047	0.5453	0.5866	0.058*	
C1	0.1884 (4)	0.58954 (18)	0.54452 (9)	0.0446 (5)	
H1	0.1923	0.5282	0.5157	0.053*	
C9	0.1163 (3)	0.97731 (17)	0.81328 (9)	0.0429 (5)	
H9	0.0615	1.0337	0.8449	0.051*	
C5	−0.0106 (3)	0.76092 (17)	0.58780 (8)	0.0423 (5)	
H5	−0.1391	0.8157	0.5878	0.051*	
C8	0.0212 (4)	1.00240 (16)	0.75099 (9)	0.0442 (4)	
H8	−0.0867	1.0724	0.7479	0.053*	
C15	0.0302 (3)	0.80528 (19)	0.88008 (9)	0.0470 (5)	
H15	−0.1075	0.8466	0.8937	0.056*	
C11	0.3875 (4)	0.79694 (18)	0.81664 (9)	0.0491 (5)	
H11	0.4905	0.8318	0.7878	0.059*	
C14	0.0830 (3)	0.6931 (2)	0.90422 (9)	0.0504 (5)	
C7	−0.0260 (4)	0.90608 (17)	0.70496 (8)	0.0397 (4)	
C12	0.4420 (4)	0.68306 (19)	0.84092 (10)	0.0567 (6)	
H12	0.5803	0.6418	0.8277	0.068*	
C16	−0.0707 (4)	0.6410 (2)	0.94879 (11)	0.0735 (7)	
C13	0.2917 (4)	0.6330 (2)	0.88396 (10)	0.0552 (5)	
H13	0.3292	0.5579	0.8999	0.066*	
N2	−0.1971 (5)	0.5975 (2)	0.98491 (12)	0.1095 (10)	

### Atomic displacement parameters (Å^2^)

**Table:**

	*U*^11^	*U*^22^	*U*^33^	*U*^12^	*U*^13^	*U*^23^
O2	0.0630 (9)	0.0466 (8)	0.0469 (8)	−0.0178 (7)	0.0058 (7)	−0.0050 (6)
O1	0.0430 (7)	0.0614 (10)	0.0576 (9)	−0.0045 (7)	0.0059 (7)	−0.0130 (7)
N1	0.0377 (8)	0.0450 (9)	0.0421 (9)	−0.0068 (7)	0.0002 (7)	−0.0096 (8)
C4	0.0367 (9)	0.0361 (10)	0.0323 (10)	−0.0069 (8)	0.0031 (8)	0.0022 (8)
C3	0.0356 (9)	0.0477 (12)	0.0457 (11)	−0.0025 (9)	−0.0070 (9)	−0.0043 (10)
C6	0.0479 (11)	0.0535 (12)	0.0344 (10)	−0.0047 (10)	−0.0047 (9)	−0.0005 (10)
C10	0.0424 (10)	0.0417 (10)	0.0327 (10)	−0.0036 (9)	−0.0020 (8)	−0.0081 (9)
C2	0.0424 (10)	0.0458 (12)	0.0561 (13)	0.0033 (10)	0.0024 (10)	−0.0049 (10)
C1	0.0531 (12)	0.0429 (11)	0.0376 (11)	−0.0061 (10)	0.0074 (9)	−0.0057 (9)
C9	0.0511 (10)	0.0401 (11)	0.0375 (11)	−0.0015 (9)	0.0085 (9)	−0.0064 (9)
C5	0.0446 (10)	0.0454 (11)	0.0370 (11)	0.0053 (10)	−0.0027 (9)	0.0009 (9)
C8	0.0537 (11)	0.0371 (10)	0.0418 (11)	−0.0009 (9)	0.0034 (9)	−0.0023 (9)
C15	0.0455 (10)	0.0526 (12)	0.0429 (12)	0.0048 (10)	0.0038 (10)	−0.0010 (10)
C11	0.0448 (10)	0.0616 (14)	0.0408 (11)	0.0010 (10)	0.0056 (9)	−0.0048 (11)
C14	0.0535 (12)	0.0542 (13)	0.0435 (12)	0.0022 (11)	−0.0021 (10)	0.0068 (11)
C7	0.0475 (11)	0.0356 (10)	0.0359 (10)	−0.0027 (9)	0.0026 (9)	0.0031 (9)
C12	0.0561 (13)	0.0611 (15)	0.0529 (13)	0.0186 (11)	0.0003 (11)	−0.0095 (12)
C16	0.0719 (16)	0.0759 (16)	0.0726 (17)	0.0088 (14)	0.0115 (14)	0.0317 (15)
C13	0.0690 (14)	0.0470 (12)	0.0496 (12)	0.0069 (12)	−0.0081 (11)	−0.0007 (10)
N2	0.1022 (19)	0.111 (2)	0.115 (2)	0.0091 (16)	0.0281 (17)	0.0571 (17)

### Geometric parameters (Å, °)

**Table:**

O2—C9	1.447 (2)	C2—H2	0.9300
O2—C8	1.448 (2)	C1—H1	0.9300
O1—C7	1.226 (2)	C9—C8	1.483 (3)
N1—C7	1.352 (2)	C9—H9	0.9800
N1—C4	1.423 (2)	C5—H5	0.9300
N1—H4	0.8600	C8—C7	1.493 (3)
C4—C3	1.384 (2)	C8—H8	0.9800
C4—C5	1.387 (2)	C15—C14	1.387 (3)
C3—C2	1.381 (3)	C15—H15	0.9300
C3—H3	0.9300	C11—C12	1.407 (2)
C6—C1	1.374 (3)	C11—H11	0.9300
C6—C5	1.385 (3)	C14—C13	1.394 (3)
C6—H6	0.9300	C14—C16	1.411 (2)
C10—C11	1.383 (3)	C12—C13	1.367 (3)
C10—C15	1.384 (2)	C12—H12	0.9300
C10—C9	1.474 (3)	C16—N2	1.155 (3)
C2—C1	1.385 (3)	C13—H13	0.9300
			
C9—O2—C8	61.64 (11)	C6—C5—C4	119.40 (18)
C7—N1—C4	125.86 (15)	C6—C5—H5	120.3
C7—N1—H4	117.1	C4—C5—H5	120.3
C4—N1—H4	117.1	O2—C8—C9	59.14 (12)
C3—C4—C5	120.47 (17)	O2—C8—C7	118.21 (15)
C3—C4—N1	118.09 (16)	C9—C8—C7	122.89 (16)
C5—C4—N1	121.43 (16)	O2—C8—H8	115.0
C2—C3—C4	119.58 (17)	C9—C8—H8	115.0
C2—C3—H3	120.2	C7—C8—H8	115.0
C4—C3—H3	120.2	C10—C15—C14	120.97 (18)
C1—C6—C5	120.21 (18)	C10—C15—H15	119.5
C1—C6—H6	119.9	C14—C15—H15	119.5
C5—C6—H6	119.9	C10—C11—C12	119.47 (18)
C11—C10—C15	119.82 (18)	C10—C11—H11	120.3
C11—C10—C9	121.80 (17)	C12—C11—H11	120.3
C15—C10—C9	118.31 (17)	C15—C14—C13	118.82 (19)
C3—C2—C1	120.03 (18)	C15—C14—C16	120.7 (2)
C3—C2—H2	120.0	C13—C14—C16	120.5 (2)
C1—C2—H2	120.0	O1—C7—N1	125.40 (18)
C6—C1—C2	120.31 (17)	O1—C7—C8	118.70 (17)
C6—C1—H1	119.8	N1—C7—C8	115.87 (16)
C2—C1—H1	119.8	C13—C12—C11	120.00 (19)
O2—C9—C10	117.57 (16)	C13—C12—H12	120.0
O2—C9—C8	59.22 (12)	C11—C12—H12	120.0
C10—C9—C8	124.29 (16)	N2—C16—C14	179.4 (3)
O2—C9—H9	114.7	C12—C13—C14	120.9 (2)
C10—C9—H9	114.7	C12—C13—H13	119.5
C8—C9—H9	114.7	C14—C13—H13	119.5
			
C7—N1—C4—C3	−143.99 (18)	C10—C9—C8—C7	1.4 (3)
C7—N1—C4—C5	37.1 (3)	C11—C10—C15—C14	−1.4 (3)
C5—C4—C3—C2	−0.2 (3)	C9—C10—C15—C14	−178.62 (18)
N1—C4—C3—C2	−179.08 (17)	C15—C10—C11—C12	1.5 (3)
C4—C3—C2—C1	−0.8 (3)	C9—C10—C11—C12	178.54 (18)
C5—C6—C1—C2	−0.8 (3)	C10—C15—C14—C13	0.8 (3)
C3—C2—C1—C6	1.3 (3)	C10—C15—C14—C16	−179.6 (2)
C8—O2—C9—C10	115.39 (18)	C4—N1—C7—O1	−2.2 (3)
C11—C10—C9—O2	3.5 (3)	C4—N1—C7—C8	179.70 (15)
C15—C10—C9—O2	−179.39 (15)	O2—C8—C7—O1	174.09 (18)
C11—C10—C9—C8	73.5 (3)	C9—C8—C7—O1	104.4 (2)
C15—C10—C9—C8	−109.4 (2)	O2—C8—C7—N1	−7.6 (2)
C1—C6—C5—C4	−0.2 (3)	C9—C8—C7—N1	−77.4 (2)
C3—C4—C5—C6	0.7 (3)	C10—C11—C12—C13	−0.9 (3)
N1—C4—C5—C6	179.55 (16)	C11—C12—C13—C14	0.3 (3)
C9—O2—C8—C7	−113.41 (19)	C15—C14—C13—C12	−0.2 (3)
C10—C9—C8—O2	−104.2 (2)	C16—C14—C13—C12	−179.8 (2)
O2—C9—C8—C7	105.6 (2)		

### Hydrogen-bond geometry (Å, °)

**Table:**

*D*—H···*A*	*D*—H	H···*A*	*D*···*A*	*D*—H···*A*
N1—H4···O1^i^	0.86	2.46	3.239 (3)	151 (1)
C11—H11···O1^i^	0.93	2.56	3.467 (3)	165 (1)
C12—H12···O2^ii^	0.93	2.55	3.302 (3)	139 (1)

Symmetry codes: (i) *x*+1, *y*, *z*; (ii) −*x*+1, *y*−1/2, −*z*+3/2.
